# Ensemble Response in Mushroom Body Output Neurons of the Honey Bee Outpaces Spatiotemporal Odor Processing Two Synapses Earlier in the Antennal Lobe

**DOI:** 10.1371/journal.pone.0050322

**Published:** 2012-11-29

**Authors:** Martin F. Strube-Bloss, Marco A. Herrera-Valdez, Brian H. Smith

**Affiliations:** 1 Max Planck Institute for Chemical Ecology, Department of Evolutionary, Neuroethology, Jena, Germany; 2 Departamento de Matemáticas y Física, Instituto de Investigaciones Interdisciplinarias, Universidad de Puerto Rico en Cayey, Cayey, Puerto Rico; 3 School of Life Sciences, Arizona State University, Tempe, Arizona, United States of America; University of Arizona, United States of America

## Abstract

Neural representations of odors are subject to computations that involve sequentially convergent and divergent anatomical connections across different areas of the brains in both mammals and insects. Furthermore, in both mammals and insects higher order brain areas are connected via feedback connections. In order to understand the transformations and interactions that this connectivity make possible, an ideal experiment would compare neural responses across different, sequential processing levels. Here we present results of recordings from a first order olfactory neuropile – the antennal lobe (AL) – and a higher order multimodal integration and learning center – the mushroom body (MB) – in the honey bee brain. We recorded projection neurons (PN) of the AL and extrinsic neurons (EN) of the MB, which provide the outputs from the two neuropils. Recordings at each level were made in different animals in some experiments and simultaneously in the same animal in others. We presented two odors and their mixture to compare odor response dynamics as well as classification speed and accuracy at each neural processing level. Surprisingly, the EN ensemble significantly starts separating odor stimuli rapidly and before the PN ensemble has reached significant separation. Furthermore the EN ensemble at the MB output reaches a maximum separation of odors between 84–120 ms after odor onset, which is 26 to 133 ms faster than the maximum separation at the AL output ensemble two synapses earlier in processing. It is likely that a subset of very fast PNs, which respond before the ENs, may initiate the rapid EN ensemble response. We suggest therefore that the timing of the EN ensemble activity would allow retroactive integration of its signal into the ongoing computation of the AL via centrifugal feedback.

## Introduction

In insects and in mammals, processing of sensory information about odors involves a series of transformations in successive stages of processing in the brain; reviewed in Wilson and Mainen [1]. To understand this processing, it is critical to understand how and why these transformations take place. Because of the relatively small size and ease of access to the brain, and because of the similarity of olfactory processing centers to that of mammals [1], insects such as the locust, moth and honey bee are excellent model animals for these questions [2]. At the first transformation, neural networks in the insect antennal lobe (AL) transform sensory input into a series of quickly evolving patterns of projection neuron (PN) activity at the output of the AL [3]–[7]. In honey bees, approximately 800 PNs make up the output of each AL, and the PN axons ‘fan out’ via different antenno cerebral tracts (ACTs) to diverge onto dendrites of the MB principal neurons, the Kenyon cells (KC) ipsilateral to each AL. Each MB consists of approximately 170,000 KCs. The different ACTs (lateral antenno cerebral tract: l-ACT; medial antenno cerebral tract: m-ACT) may be functionally optimized for parallel processing of complex olfactory information, possibly reflecting different ecological demands in Hymenoptera [8].

At the input to the MB, each PN axon diverges onto synaptic contacts with several different KCs. Thus each KC receives and summates coincident input from many different PNs [9]. The KCs have three critical properties that govern their function. They are mostly silent at rest [3], [10]. They require coincident inputs from several PNs in order to fire [3], [11]. Finally, when they fire they reset to quiescence quickly due to recurrent inhibition [12], [13].

At the next synapse, KC axons then converge onto dendrites of approximately 400 extrinsic neurons (ENs) around the MB alpha-lobes [14]–[17]. ENs like the antennal lobe feedback neuron AL-1 [17], [18] or the ALF-1 neuron [16] connect the MB output retrograde with the AL. They have been related to the centrifugal system following the terminology of their analogs in vertebrates [19]–[21]. In naive animals ENs respond rather generally to many different odors. But after classical odor conditioning they encode the odor reward association, which is established at the MB output already 140 ms after stimulus onset [22]. Thus, after conditioning the MB quickly separates the rewarded stimulus from other odor stimuli.

We designed a series of experiments to assess how divergent (AL to MB) and convergent (MB to EN) connectivity may play a role in both speed and accuracy of odor classification. To assess this question we performed in vivo recordings from both the PNs of the AL and the ENs of the MB in the honey bee, either in different animals or simultaneously in the same animal. Using these recordings, we compared the differences among olfactory representations of two pure odorants and their binary mixture at both stages of processing. We found that at the MB output the EN ensemble separated the input stimuli faster than the PN ensemble at the AL output, which implies that in general the connectivity between the AL and the MB may increase the speed of odor classification rather than classification performance itself. Thus at the MB output a rapid “first impression” of the odor stimulus is established at a time point at which the AL network still has not reached its maximum odor separation. This very rapid stimulus representation might allow for integration of the MB output into the ongoing AL computation via feedback, which could be facilitated by an identified anatomical centrifugal pathway.

## Materials and Methods

### Animal

**Figure 1:**
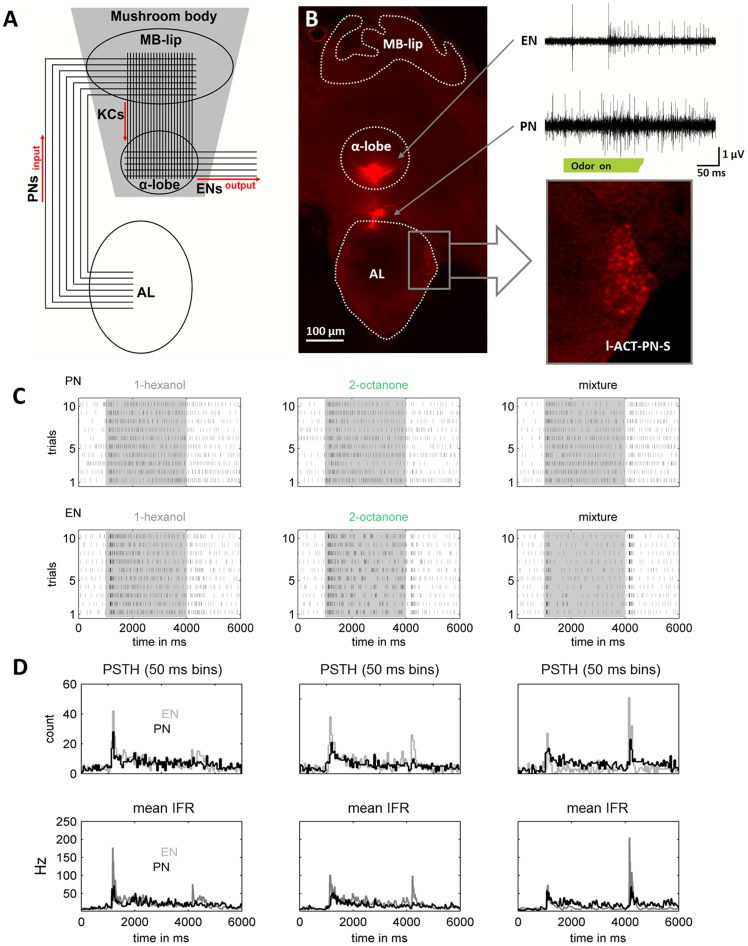
Simultaneous extracellular recordings of the input and the output of the mushroom body (MB). (**A**) Divergent-convergent olfactory processing in the mushroom body (MB). Divergence: around 800 projection neurons (PNs) send information of the Antennal lobe (AL) to the Kenyon cells (KC) of the MB lip (∼170,000 KC per MB). Convergence: at the MB output ∼400 extrinsic neurons (ENs) read out activity from the KC axons. The red arrows indicate the assumed direction of neural excitation flow. (**B**) Confocal microscope image of the recording electrode positions. One electrode was inserted into the ventral part of the alpha-lobe, the other electrode into the dorsal rim of the AL. **Zoom**; Somata of lateral antenno-cerebral tract projection neurons (l-ACT-PN-S) were back-filled with dye that coated the electrode tips [16]. Differential recording combinations from the three wires of each electrode revealed signals showing nicely defined wave shapes of different extracellularly recorded action potentials from ENs (top) as well as from PNs (bottom). (**C**) Dot displays of a simultaneous recorded PN-EN couple in response to 1-hexanol, 2-octanone and the mixture. (**D**) Upper raw: peri stimulus time histograms (PSTH's, 50 ms time bins); lower raw: mean instantaneous firing rates (IFR, in ms) of the PN-EN couple in (C) (PN = black, EN = gray). Note, the dot displays, the PSTHs and the mean IFRs reflect the same odor response dynamics.

No specific permits were required for the described field studies. All experiments used honey bees maintained in a standard breeding program to control genetic variation among animals. Pollen foraging honey bees (*Apis mellifera*) were caught at the entrance to the hive in the morning two hours before the experiment. Preparation of the bees as well as electrode positioning have been previously described [22]. To record the activity of mushroom body extrinsic neurons (ENs) one electrode was inserted at the ventral border of the alpha-lobe (Figure 1B and Figure S1) where ENs have massive axons, which allows a separation from their extracellular recorded activity from KC activity (cp. supplemental material in [22]). To record activity from PNs, electrodes were inserted at the dorsal rim of the AL (Figure 1B and Figure S1). We recorded PNs and ENs in 20 bees respectively. In a subset of 8 animals we recorded both stages simultaneously. Overall we analyzed 111 PNs and 75 ENs out of 48 bees.

### Visualization of the recording position

Before inserting, electrodes were dipped in a fluorescent dye (DiI, invitrogen.com), to allow visualization of the electrode positions relative to the neuropiles of the brain (Figure 1B and Figure S1). After the experiment the electrodes were removed, brains dissected and fixed in 4% formaldehyde diluted in 50% methanol for 24 hours at 4°C. Further on brains were rinsed for 20 minutes in phosphate-buffered saline (PBS; pH 6.7), diluted 1∶4 in distilled water, dehydrated in an increasing ethanol series (30%, 50%, 70%, 90%, 99%, 100%, 10 minutes each), cleared in a mixture of 50% methyl-salicylate (MS) and 100% ethanol and embedded as whole mounts in MS in double-sided custom slides. We used a confocal laser scanning microscope (Leica TCS SP2) with a Leica HC PL Apo ×10/0.4 dry lens objective to scan the brains under blue excitation and green emission light.

### Odor stimulation

We used the same type of olfactometer as described elsewhere [22]. Two odors (1-hexanol and 2-octanone, Sigma-Aldrich Chemie GmbH) and their mixture (1∶1) were presented 10 times each in a pseudorandomized sequence with inter-stimulus intervals of 30 seconds. Both odors evoke very distinct glomerular activity in honey bees [7]. The duration of odor stimulation was always 3 seconds. The odors were diluted in Paraffin oil 1∶100. Each odor chamber of the olfactometer contained a filter paper strip (1×1 cm) loaded with 10 µl of the odor/mineral oil solution for the single components. The filter paper of the chamber charged with the mixture was loaded with twice the amount (20 µl) to use a comparable amount of the single components in the mixture and the pure odor. Odors were injected into a constant air stream (1.5 m/s speed) which was delivered through a Teflon hose (10 mm in diameter). During the three-second odor stimulation, only half (2.5 ml) of the odor chamber air volume was injected into the constant air stream via an injection needle (0.5 mm in diameter) to avoid concentration gradients.

### Electrophysiology and data preprocessing

All of the recording techniques have been described in detail by Strube-Bloss [22]. In short: self-made three channel extracellular recording electrodes were mounted on a micromanipulator (World Precision Instruments PM5500). In order to extract the local spiking activity, we used a 16 channel analog recording system (Neuralynx, Bozeman, MT, USA) to make differential recording combinations out of the single wires of each electrode. A silver wire with a diameter of 25 µm (Nilaco, Tokyo, Japan) was inserted into the right compound eye and served as a reference electrode. Signals were recorded with 30 kHz and pre-filtered (300 Hz) before storage. We used a software high-pass filter (over 800 Hz) before we applied a semi-automatic spike sorting technique (template-matching) in Spike2 (Cambridge Electronic Design, Cambridge, UK) described in the supporting information (Figure S1). Throughout the text we use the term ‘extrinsic neuron’ (EN) and ‘projection neuron’ (PN) in synonym to the term ‘unit’ to improve readability and to stress the fact that we recorded and sorted spiking activity from mushroom body extrinsic neurons and from projection neurons. The Spike trains were imported in MatLab for further analysis.

### Single unit and population response

For each trial instantaneous firing rates (iFRs) were obtained by calculating the inverse of the recorded inter spike intervals. To reach millisecond resolution in each trial we calculated an interpolated version of the iFRs (IFR) using cubic splines with a time step of 1 millisecond. The data was further aligned around the stimulus onset of the 10 repetitions per odor and used for calculating a time series representative for the average instantaneous firing rate (mean IFR) of each unit.

To extract the odor classification into the different neuronal ensembles we calculated Euclidean distances (*L*2-Norm). For an ensemble of *n* neurons and a given stimulus *a*, we constructed the n- dimensional population vector (*v^a^*) using the mean IFRs of each unit. We than used the population vectors of two stimuli, **a** and **b** and calculated their distance at each point in time as follows; *d(t)  =  (Σ(v_i_^a^(t) – v_i_^b^(t))^2^)^1/2^*, where *i* is an index for the i-th neuron.

In addition to the Euclidean distances we used principal component analysis (PCA) to visualize the population activity of the two neuronal ensembles we recorded from. We again used all the IFRs of the single units of each ensemble. To separate the main contributing units for the different odor representations we performed PCA separately for each neuronal class and each odor using the MatLab statistics toolbox. To keep the temporal aspects of the ensemble activity intact, PCA was performed taking into account time as the source of sample points, and number of neurons as the dimension of the original component space. The first three principal components (PC1, PC2 and PC3) were used to compare the time courses of the ensemble responses. Further on we used PCA to visualize the odor separation at each neural population level. Therefore we performed PCA on a matrix which reduced again the number of neurons as the dimensions of the original component space and kept the temporal aspect of the ensemble activity across the different odor stimuli intact.

### Statistical analysis

To compare the distributions of IFRs between ENs and PNs and between the different odor stimuli on each single population level we used a nonparametric version of the Mann-Whitney U-test (Wilcoxon ranksum test). In addition we used the Kolmogorov-Smirnov test to compare distributions of Euclidean distances between the PN and the EN ensemble. To analyze the time course of IFRs relative to stimulus onset times, we assumed that significantly high firing rates in each neuron are indicative of a phasic response to the stimulus. To find significantly high firing rates for each neuron, we set a significance level q, and found the qth-percentile of the IFR distribution [23], [24]. The firing rate associated with q was used as a threshold beyond which the firing rates were deemed as significantly high (Figure S2). Significantly high firing rates were found by taking into account all firing rates displayed by each neuron during the recording, including spontaneous activity, and times around stimulus presentations. Different values of q were tested to assess the sensitivity of our analyses and the quality of our interpretations. We defined the On-response latency as the start time of an interval between 10 and 500 milliseconds post-stimulus onsets during which the firing rate was significantly above baseline. Single unit response latency's were calculated in each single trial using the IFRs and averaged over trials in which a response was detected (up to 10 trials per unit).

To calculate the latency of the odor separation into the different neuronal populations we used the Euclidean distances calculated as described above. The significance level q of the ‘thresholding’ algorithm described above was used to find the times at which the distance **d** took significant values (q>0.95). In this case, a distribution was obtained by sampling different values of **d** over time, in order to find the minimum time at which the first significantly large value of **d** was obtained (population response latency).

## Results

### Recording simultaneously from PNs and ENs

**Figure 2:**
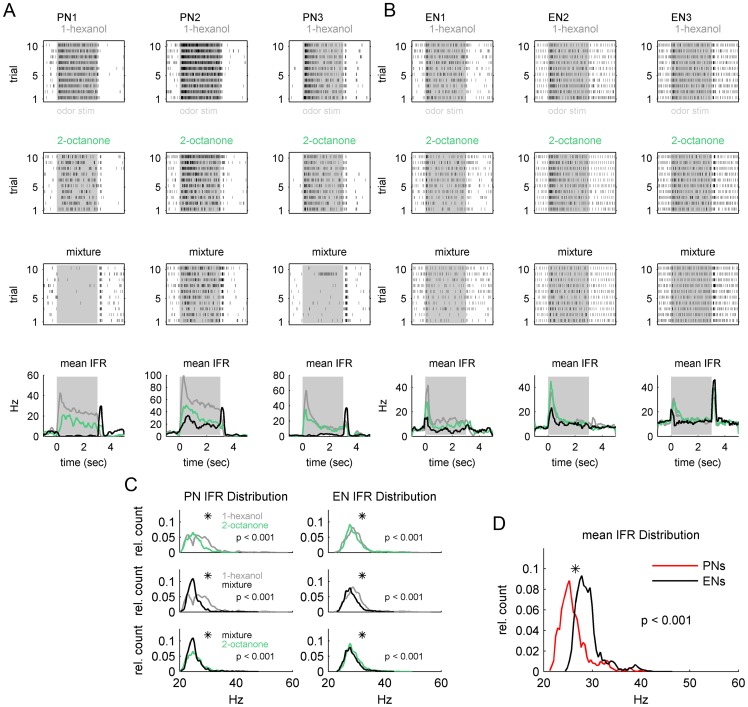
Distinct Instantaneous Firing Rate (IFR) distributions in PNs and ENs. (**A**) Examples of three typical PN-units (columns). Each column includes the dot displays for the different odors consisting of 10 repetitions (trials) were each dot corresponds to one action potential. At the very bottom of each column the mean IFR is shown (grey  = 1-hexanol, green  = 2-octanone, black  =  mixture). Odor stimulation is marked in grey. (**B**) Same as in (A), but for three typical EN-units. Both, PN- and EN-units show on and off responses, which were relatively sharp in the EN-units. In addition a tonic component appears to be very prominent in the PN-unit responses (A). (**C**) IFR distribution functions (IFRs) of all PN-unit's (N = 111) and all EN-units (N = 75) broken down by odor (color code same as in A). In both, PN-units and EN-units IFRs were significantly different (Wilcoxon rank sum test: p<0.001) for all odors. (**D**) Averaged across odors the mean IFRs clearly separate the recorded PN-ensemble from the recorded EN-ensemble (Wilcoxon rank sum test: p<0.001). Note, the IFRs of the PNs were dominated by the stimulus outlasting tonic response component, whereas EN IFRs reflecting the phasic activity around stimulus on- and off- set.

We recorded neuronal activity at two stages of olfactory processing: Before the anatomically divergent PN-to-KC pathway and after the anatomically convergent KC-to-EN pathway (Figure 1A and 1B). In total we analyzed 111 PNs and 75 ENs. Each of two single odor components and their mixture were presented 10 times respectively for a total of 30 presentations (dot plots: Figure 1C, Figure 2A and 2B). From the 10 repetitions of each stimulus we calculated peri-stimulus time histograms (PSTHs, bin size  = 50 ms) and mean instantaneous firing rates (IFRs, ms resolution) for each unit (Figure 1D). Both measures accurately reflected the response dynamics illustrated by the dot displays for each unit. As described in the materials and methods section, the IFRs allowed us to analyze the data with millisecond resolution.

### Units recorded from AL and MB outputs are distinct and differ in response

The units that we recorded at each site represent distinct populations with different characteristic firing patterns. These respective patterns for PNs and ENs are consistent with units recorded in the AL and MB in the locust [25], [26] and the honey bee (AL: [5], MB: [27], [28], [29]). Both neuronal types responded with combinations of odor on- and off- responses (Figure 2A and 2B). In addition, at the PN level we often observed tonic excitation throughout the stimulus duration which was less pronounced at the EN level (cp. examples in Figure 2A and 2B).

The IFR we calculated for each unit and odor as shown in figure 2A bottom was used to calculate the mean IFR distribution functions for the PN population (N = 111) and the EN population (N = 75). Broken out by odor, the IFR distribution of both the PN and EN ensembles were significantly odor dependent (Figure 2C; Wilcoxon rank sum test, p<0.001) whereat ENs showed less dramatic changes in their distribution function (Figure 2C). Next we calculated the averaged IFR distribution across all odor stimuli of all PNs and all ENs respectively. Both were significantly different (Wilcoxon rank sum test, p<0.001, Figure 2D). The distribution of the EN ensemble showed its maximum at about ∼28 Hz. Whereas the IFR distribution function of the PN ensemble showed a mode at a lower rate ∼24 Hz. Thus, the IFR distribution functions support that we were recording from two distinct populations of units.

### Transient and fixed point dynamics in the AL network

**Figure 3:**
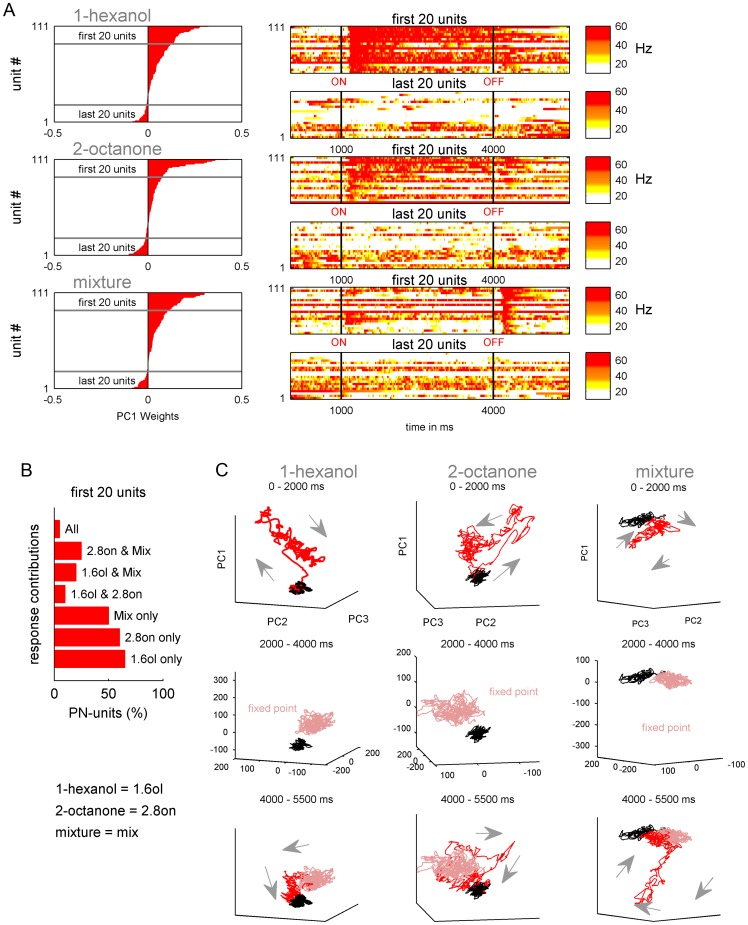
Principal Component Analysis (PCA) of the PN ensemble for each odor stimulus. (**A**) left: The weights of each unit (red bars; N = 111) on the first principal component (PC1) was used to order the units from positive (top) to negative weights (bottom). (**A**) right: the first (positive weights) and the last (negative weights) 20 units were used to illustrate the features extracted by PC1. Each line represent the false color coded IFR calculated out of the 10 repetitions per odor and unit. Stimulation is marked by black lines. PC1 contrasted odor sensitive (positive weights) and insensitive units (negative weights). (**B**) Most of the PN units were stimulus selective. For example; from the 20 first main contributing units of 1-hexanol, 65% respond to 1-hexanol only, 10% to 1-hexanol and 2-octanone, 20% to 1-hexanol and the mixture, and 5% to all stimuli. The same tendency was shown by the first 20 main contributing units of 2-octanone. (**C**) Projection of the transients of the first three PCs (PC1, PC2 and PC3). The column's separate the odors while the rows represent different time windows during stimulus presentation. Background activity of 1000 ms before odor onset is marked in black. The first row illustrates the first 1000 ms after odor onset (red), during which time there is a rapid divergence from background to form a transient. During the following 2000 ms (second row) odor evoked activity (pink) was clearly separated ‘fixed point’ from the spontaneous activity before odor onset (black). During the 1500 ms following the odor off set (third row, red) the activity returns to the background (black).

We applied principal component analysis (PCA) to the PN ensemble activity for each odor stimulus separately (Figure 3). We used the contribution (weights) of each unit to the first principal component (PC1) to describe the features of the response extracted by the PCA (Figure 3A). In each case, PCA described a contrast between PNs that were activated by a given odor (positive weights) and those that were not (negative weights). PNs that had negative loadings failed to show a consistent change in response at odor onset. We used the 20 units showing the largest positive weights per odor and calculated their overlap. From the 20 first main contributing units of 1-hexanol, 65% respond to 1-hexanol only, 10% to 1-hexanol and 2-octanone, 20% to 1-hexanol and the mixture, and 5% to all stimuli. The same tendency was shown by the first 20 main contributing units of 2-octanone (Figure 3B). Overall the activated PN-units respond rather specific to 1-hexanol, 2-octanone or the mixture. Relatively fewer units responded to any other combination of the components and/or the mixture.

We then used the first three PCs (PC1-3) to visualize the odor evoked trajectories of each population response (Figure 3C). All of the odors evoked a clear separation of odor-evoked activity from baseline activity recorded before odor onset. During presentation of the single odor components (1-hexanol and 2-octanone) the most dynamic change in activity occurred during the first 1000 milliseconds after odor onset (first row of graphs in Figure 3C). For both odors there was a rapid shift from background activity (first arrow away from baseline), which was immediately followed by a slightly slower shift in activity (remaining arrows). In comparison, the initial activity during the mixture presentation was less pronounced compared to the single components. After the initial on-transient, the activity patterns for all of the odors settled into a relatively stable ‘fixed-point’ over the remaining 2000 ms of odor stimulation (middle row of graphs in Figure 3C). Importantly, this fixed activity remained distinct from the initial background activity, and corresponded to the tonic portion of the PN responses.

Another distinct transient activity pattern resulted when the odor was switched off (Figure 3C, bottom row). These off-transients reflected a temporary increase in PN ensemble activity during the transition from the relatively stable fixed activity to the original background activity level. As with the on-transients, the off-transients differed for each odor. For example, the off-transient to the mixture was much more pronounced than for either of the components.

### Sharp odor on- and off- transients at the MB output

**Figure 4:**
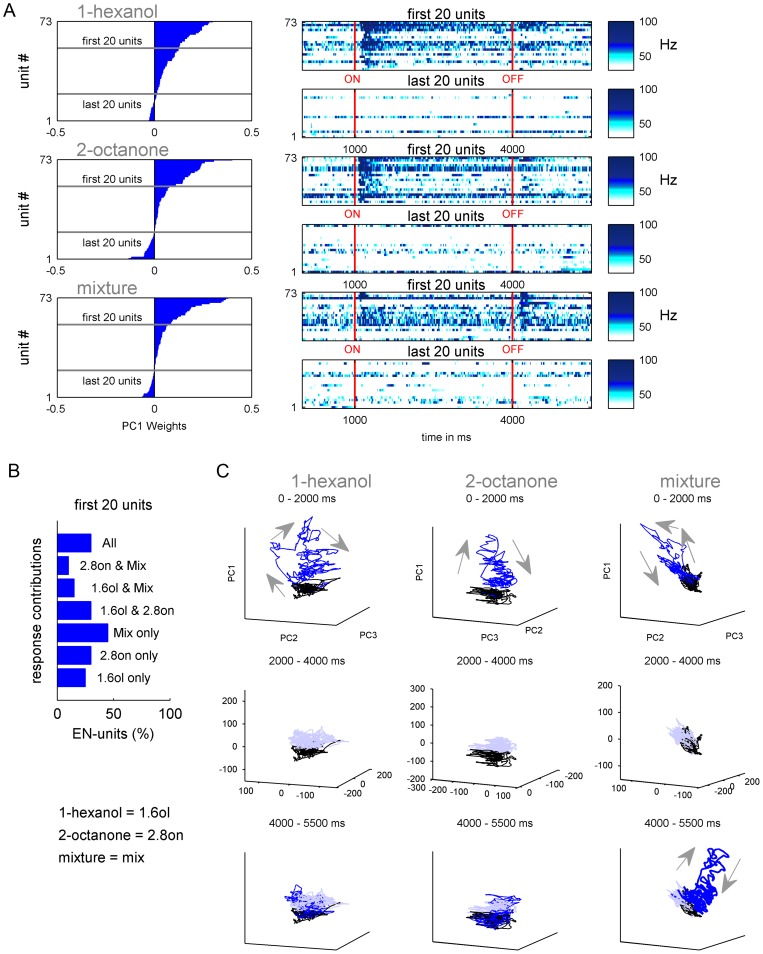
Principal Component Analysis (PCA) of the EN ensemble for each odor stimulus. (**A**) left: The weights of each unit (blue bars; N = 73) on the first principal component PC1 was used to order the units from positive (top) to negative weights (bottom). (**A**) right: The first and the last 20 EN-units were used to illustrate the features covered by PC1. Each line represent the false color coded IFR calculated out of the 10 repetitions per odor and unit. Stimulation is marked by red lines. PC1 contrasted odor sensitive and insensitive units. (**B**) In contrast to PNs (Figure 3), the response spectrum of the ENs is rather odor unspecific. Most of the 20 main contributing units were involved in the representation of at least two stimuli. For example from the 20 first main contributing units of 1-hexanol, 25% respond to 1-hexanol only, 30% to 1-hexanol and 2-octanone, 15% to 1-hexanol and the mixture, and 30% to all stimuli. The same tendency was shown by the first 20 main contributing units of 2-octanone. (**C**) The transients of the first three PCs (PC1, PC2 and PC3) were drawn. The column's separate the odors while the rows represent different time windows during stimulus presentation. Background activity of 1000 ms before odor onset is marked in black. The first row illustrates the first 1000 ms after odor onset (blue), during which time there is a rapid divergence from background to form a transient. In contrast to PNs there is no ‘fixed point’ dynamics during the following 2000 ms (second row, light blue). During the 1500 ms following the odor off set (third row, blue) the most drastic activity increase occurs for the mixture.

We applied the same analysis to the EN responses (Figure 4). ENs that had high positive weights showed strong phasic activity to 1-hexanol, 2-octanone and to the mixture (Figure 4A). Negative loadings for all three odors indicated a lack of response. In contrast to the PNs, relatively few ENs were specific to only one odor. Most of the ENs were involved in the representation of at least two odors (Figure 4B). This rather broad response profile is consistent with the literature [22], [29], [30].

The trajectories of the first three PCs show that all of the odors evoked a clear separation of the activity from background during the first 1000 milliseconds after odor onset (first row of graphs in Figure 4C). For all odor stimuli there was a rapid shift from baseline activity. This reflects the strong on-transients typically observed in EN recordings. In contrast to the PN responses, the EN response patterns collapsed back to approximately baseline during the remaining 2000 ms of odor stimulation (middle row of graphs in Figure 4C). Therefore, a distinct ‘fixed point’ in the PN ensemble, corresponding to a sustained tonic response of PNs, was not observable in ENs. A sizable transient was observed once again when the odor was switched off (Figure 4C, bottom row). These off-transients reflect a temporary increase in EN ensemble activity and, as with the PN ensemble, they were most prominently observed in responses evoked by presentation of the mixture. Thus, the activity of the EN ensemble is characterized by responses to odor on- and/or off-set, but low responsiveness to odor presence per se.

### Rapid odor classification at the MB output

**Figure 5:**
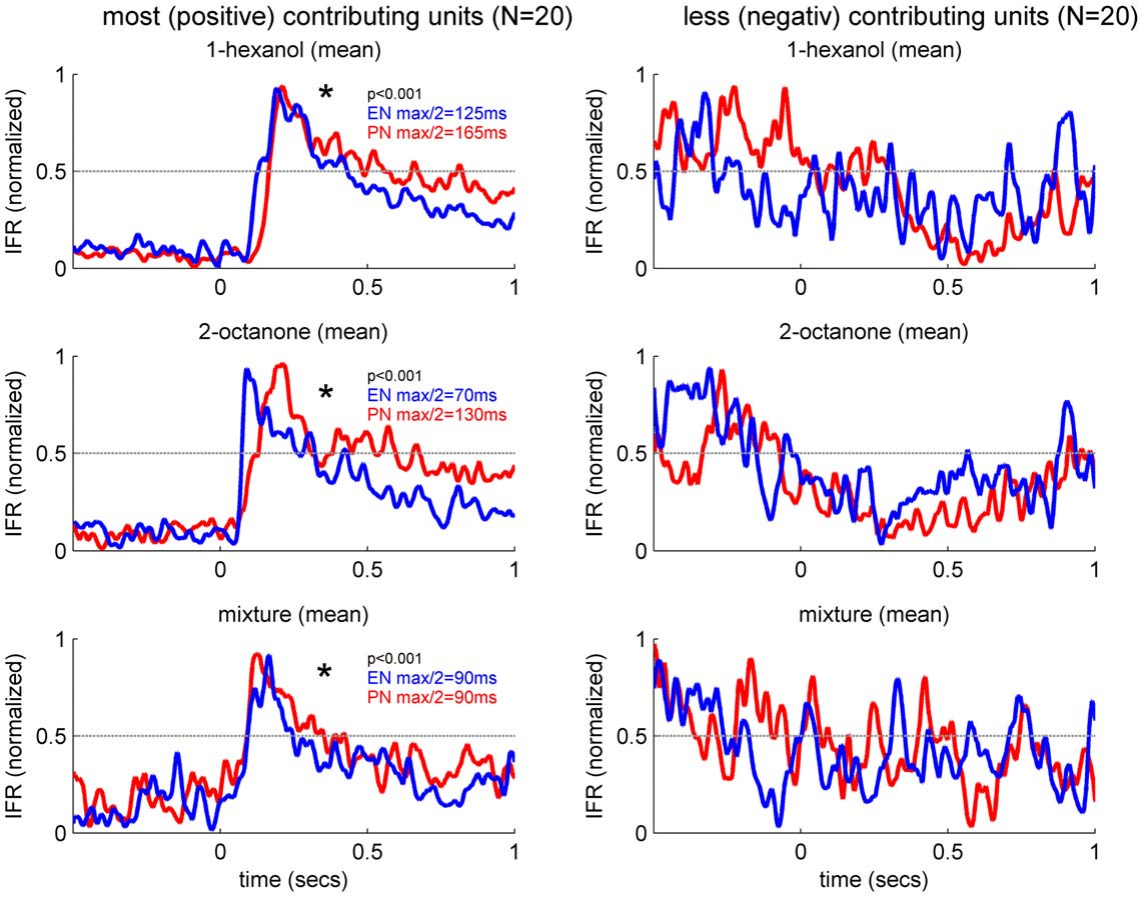
Rapid excitatory activity in response contributing ENs. Normalized mean instantaneous firing rate (IFR) for the different odor stimuli. (**Left**) IFR of the first 20 PN-units (red, cp. Figure 3A) and the first 20 EN-units (blue, cp. Figure 4A) showing the most positive contributions to the related odor representation. For the pure odors 1-hexanol and 2-octanone the mean EN responses reached the half maximal response rate 40–60 ms before the PN-units. Before normalization the mean rates of PN-units and EN-units were tested using a Kolmogorov Smirnov test (p<0.001). Mean rates in PNs and ENs were significantly different for all stimuli. (**Right**) IFR of the last 20 PN-units (red, cp. Figure 3A) and the last 20 EN-units (blue, cp. Figure 4A) showing the most negative contributing units. Note, there was no odor related activity in units contributing negatively to the odor representation.

Next we compared the timing of the population responses at the AL and MB outputs. For a first quick impression we compared the average time to reach half maximum mean firing rate for the 20 PNs (Figure 3A) and 20 ENs (Figure 4A) that contributed most to the representation of each odor (Figure 5, left). Interestingly the normalized average IFRs revealed that the EN population responded as fast or even faster to the odors compared to the PN population. The mean time to reach half maximum firing rate for the ENs was faster than for the PNs for 1-hexanol and 2-octanone, where the PNs trailed the ENs by 40 ms and 60 ms respectively. For the mixture the times to reach half maximum were equal. The averaged firing rate for the 20 PNs and 20 ENs contributing most negatively to the representation of each odor showed no inhibitory or any odor related activity (Figure 5, right).

**Figure 6:**
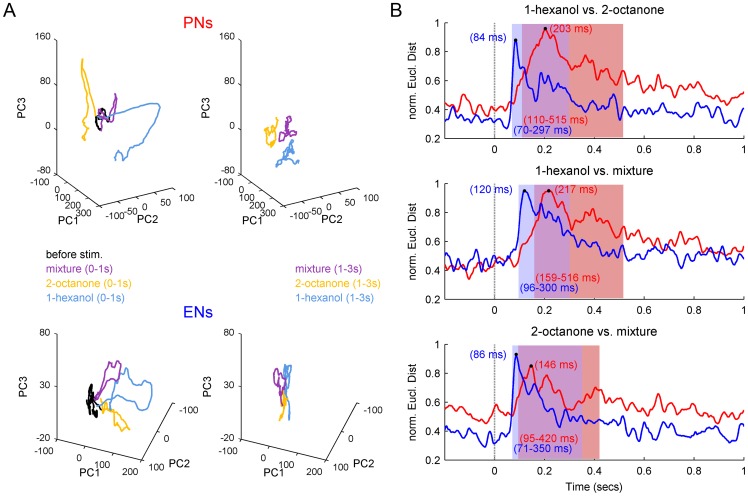
Ensemble response in ENs outpaces odor processing in the AL. (**A**) Visualizing the odor separation in both neural ensembles using principal component analysis (PCA) illustrates that at both levels odors were separated. Plotting the first three PCs (PC1, PC2 and PC3) let appear three odor dependent trajectories (1-hexanol in blue, 2-octanone in yellow, mixture in violet). However, the PN-ensemble maintained the separation throughout the odor presentation (stimulation second 1–3, top right) whereas in the EN-ensemble the initial odor separation during the first second of odor presentation (bottom left, 0–1s) collapsed to the background activity before odor onset (black) during the last two seconds of odor presentation (bottom right, 1–3s). (**B**) In order to analyze the timing of odor separation we calculated the Euclidean distances between the population vectors for the different odor representations at each neural level (PN-ensemble in red, EN-ensemble in blue). Distances were normalized. The first second after stimulus onset (grey dashed line) is shown. Red and blue boxes (with ranges shown) marking the time windows during which the Euclidian distances for the PN and EN ensemble were significantly distinct from background (significance level q>0.95). Significant odor separation of the three possible combinations of stimulus pair's occurs faster in the EN-units (blue) than in PN-units (red). EN-units also always reached their separation maximum (black dots) before the PN-units (numbers given in the panels). In addition the odor separation last longer in the PN-unit network for all odor combinations.

In the next step we focused on the odor separation at both levels. To visualize the odor separation in each neural population we reduced the data using Principal Component Analysis (PCA), which represented the ensemble activity as a trajectory using the first three principal component axes (PC1-3). At both neural levels the three odor stimuli evoked distinct trajectories (Figure 6A, left). However, the PN ensemble sustained separation of the odors longer than the ENs because of the tonic phase of the PN firing pattern at or around the “fixed point” activity, which was absent in ENs (Figure 6A, right). Next we asked whether the peak separation of PNs and ENs differed. To do so we calculated the Euclidean Distances between the different odor representations at each time point using all 111 PNs and 75 ENs (cp. materials and methods). For all three pairs of odors the maximum separation reached by the EN ensemble never exceeded the maximum separation by the PN ensemble (Figure 6B). However, the odor separation in the EN ensemble always reached significance before the PN ensemble (significance level q>0.95). For example, the EN ensemble significantly separated 1-hexanol and 2-octanone ∼70 ms after odor onset (Figure 6B, top). The PN-ensemble followed 40 ms later at ∼110 ms after odor onset. The EN ensemble also reached a peak separation earlier than the PN ensemble. For this pair of odors the EN ensemble reached a peak separation ∼84 ms after odor onset whereas the PN ensemble reached peak separation at ∼203 ms. The separation between 1-hexanol or 2-octanone and the mixture shows the same phenomenon, namely the EN ensemble separated the odors faster than the PN ensemble (1-hexanol vs. mixture: ENs  = 96 ms, PNs  = 159 ms; 2-octanone vs. mixture: ENs  = 71 ms, PNs  = 95 ms; Figure 6B). Furthermore the significant odor separation by the PN ensemble (significance level q>0.95) always last longer compared to the EN ensemble.

### Early (fast) PNs initiating the EN ensemble response

**Figure 7:**
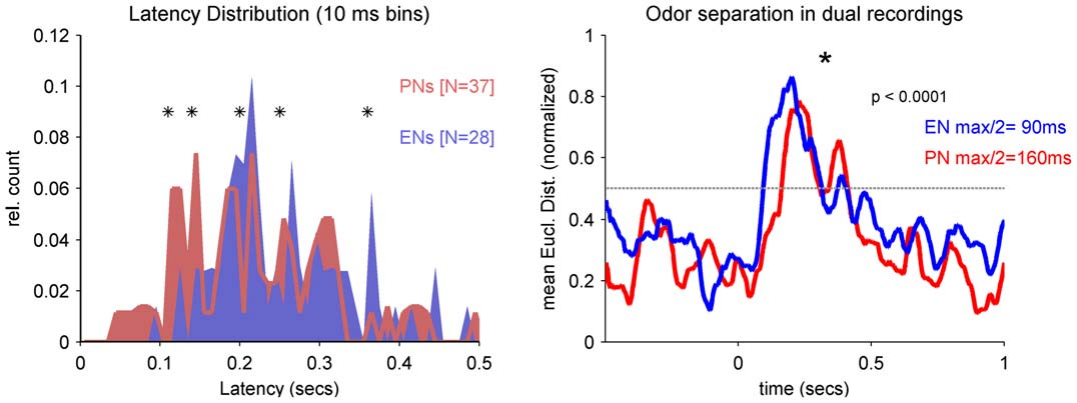
Fast PNs mediate the rapid EN ensemble response. (**A**) The latency distribution of 37 PNs (pink) and 28 ENs (blue) out of the bees in which we recorded both levels simultaneously are shown independent of odor identity. Asterisks indicate the significant distances between both distributions (significance level q>0.95). The distribution of the PN latencies starts at ∼40 ms. At this time no EN has responded. The EN latency distribution starts at ∼80 ms. In addition there are significantly more PNs starting between 100–140 ms. At around 200 ms after stimulus onset this relationship flipped and there are significantly more ENs starting their responses. (**B**) Averaged Euclidean distances calculated out of the three odor pairs (1-hexanol vs. 2-octanone, 1-hexanol vs. mixture and 2-octanone vs. mixture) for PNs (N = 37, red) and ENs (N = 28, blue) which were recorded simultaneously in 8 bees. Distances were normalized and the half maxima (grey dotted line) are drawn. Note, also in the reduced dataset of 8 bees the EN-population separates the odor stimuli faster than the PN-population (cp. Figure 6B).

We did not anticipate the relatively faster response time and classification of the EN ensemble, given that ENs receive synaptic drive from PNs via the Kenyon Cells. We therefore evaluated whether the EN ensemble might be driven by a few key PNs, which would not necessarily be revealed by the analysis with the entire PN ensemble. We concentrated on relative response latencies and evaluated the overall latency distribution of simultaneously recorded PNs and ENs (odor independent). The distribution functions illustrating that on average in early phases (∼40–150 ms) of the odor response more PNs starting their activity whereas in later phases (∼200 ms) significantly more ENs starting being active (Figure 7). The earliest PNs start to respond at ∼40 ms whereas the earliest ENs start at around ∼80 ms. Thus, although the population response of the PNs is prolonged (Figure 3C and Figure 6) the fastest PNs may trigger EN activity. In all 8 bees in which we recorded PN and EN activity simultaneously we found a PN responding before an EN (Figure S3). In addition the individual latency in relation to the population response in both, PNs and ENs can vary dependent on the odor.

## Discussion

The focus of our study was to be able to draw conclusions about the functions of transformations in successive stages of processing in the brain. We therefore recorded the activity of PNs of the AL, representing the output of the first order olfactory processing center, and the ENs of the MB, representing the output of a higher order multimodal integration center in honeybees. Our results suggest that at the MB output a very rapid “first impression”, implying a classification, of the odor stimulus is formed when the animal is naive about the meaning of the odor. Surprisingly, the classification at the output of the MB is faster than the classification reached by the spatiotemporal activity at the output of the AL, although it is two synapses earlier in processing. This finding opens new perspectives about the role of the MB as a higher order multimodal integration and learning center.

### Rapid rather than better odor classification at the MB output

The output of the honey bee AL is represented by ∼800 PNs (Figure 1A). PN axons project out of the AL along two primary tracts, the m- and the l-ACTs [14], [15] which may carry different information about the present odor stimulus [8], [31]. However, using extracellular recordings we could not assign PNs to one or the other tract. Further intracellular recordings and staining might be necessary to evaluate this question. PN axons from the two olfactory tracts diverge onto the dendritic fields of KCs forming the lip of the MBs calyx. Other modalities were received by KCs of the collar and the basal ring [14], [32], [33]. However, axons of all KCs (∼170,000 per calyx) project ventrally to form the peduncle and different output regions of the MB, like the alpha-lobe, where they converge onto ∼400 partially multimodal extrinsic neurons (ENs) [17]. The convergence of inputs from many PNs that respond to different odors onto any given EN probably underlies the changes in response tuning between PNs and ENs (Figure 3B and Figure 4B). PNs tended to be more specialized and respond specifically to one of the more basic odors, which were the two pure odors and the binary mixture. In contrast ENs responded more often to multiple odors.

Theoretically, this pattern of connectivity should also improve classification of odor stimuli by, in the first step, a nonlinear transformation from the AL PN patterns to a higher number of sparse patterns into the MB KCs and, in a second step, a linear classification of KC activity by MB ENs [34], [35]. However, our results suggest that this divergent-convergent connectivity scheme does not necessarily increase odor separation. Instead it facilitates faster odor separation, since the odor separation into the EN population (convergence) was always faster (24–64 ms) compared to the odor separation into the PN-population (divergence) (Figure 6B).

Any discussion of this faster separation in the ENs needs to be predicated on an understanding of the different spatiotemporal response patterns in the PNs and ENs. In PNs a very fast high-frequency on-response was typically followed by a constant tonic pattern of firing (Figure 2). The combination of excitatory phasic-tonic responses is stereotypical in honeybee PNs [4], [36]–[38], and it has been commonly observed in *Drosophila* [39]–[41], in the silkmoth *Bombyx mori* [42] and in the locust *Schistocerca americana* [25], [43]. The same stereotypic response dynamics have also been reported for mitral cells in the olfactory bulb of the zebrafish *Danio rerio* [44], [45]. This produced a fast ‘transient’ change from baseline activity that continued for the duration of the stimulus. This pattern has been described for locusts, where the spatiotemporal transient response reaches a stable ‘fixed point’ after approximately one second of stimulation [25]. Our data suggest the same kind of a fixed activity established in the PN ensemble around one second after odor onset (Figure 3C and Figure 6A).

In contrast, single unit activity of the recorded ENs showed mostly phasic on- and off-responses to odor stimulation (Figure 2B and Figure 4C). The tonic component throughout the odor presentation is less pronounced. These typical dynamics of ENs were also reported from previous recordings [27]–[30]. This pattern is consistent with the KC activity, which typically show sparse phasic on and often off responses to odor stimulation [46], [47], [48]. The fast, phasic responses of KCs occur because the initial activity is quickly shut down by recurrent inhibitory feedback [12], [13]. The summed information of many sharp KC on's and/or off's may drive the EN activity at the MB output and would explain their rapid phasic spiking behavior.

### A subset of rapid PNs may cause rapid odor separation at the MB-output

An important implication of our results is that at the MB output the speed of odor classification is increased compared to the odor separation in the PN population at the output of the AL. This result is surprising, because these outputs are at least two synapses away from the PN outputs of the AL. However, in simultaneous recordings at both stages we found a relatively fast subpopulation of PNs. Latencies of the fastest PNs occurred around 50 ms after odor onset. This is consistent with the population response of an intracellular recorded subset of PNs (N = 17) recorded in honey bees, which was significantly increased 54 ms after odor onset [49]. These rapidly responding PNs could serve to initiate the EN ensemble response via the KCs. This fast excitatory PN response behavior is different compared to PN responses in *Manduca sexta*, were the PN population response decreased 100–140 ms after odor onset before reflecting excitatory activity starting 120–140 ms after odor onset [50]. Each KC receives input from many different PNs reflected by their intracellular recorded sub threshold activity. For example, in the locust each KC is connected to ∼50% of neurons of its input population [9]. Converging coincident inputs of several PNs firing within a restricted temporal window, such as the earliest responding PNs in our study, could be what caused the KCs to fire action potentials. Indeed, a previous calcium imaging study in honey bees showed that the average response delay in KCs is shorter than in PNs [47]. The typically sparse phasic responses of KCs [46], [47] is probably summed up after convergence by the ENs and may be the cause for their rapid and precocious ensemble response resulting in an odor separation 70–96 ms after odor onset (Figure 6B).

The speed of classification is consistent with behavioral conditioning studies with odors. In honey bees, the expression of a conditioned response occurs regularly by 400–500 ms after odor onset [22], [51]. This latency is consistent with the fast classification of an odor by the EN ensemble, which we have identified here, given that premotor integration and initiation of an excitatory response in the musculature can easily take a few hundred milliseconds.

### Rapid classification as prerequisite for integration and/or feedback

It is therefore clear that the evolving PN response continues for the length of the stimulus and lasts long after successful EN classification and a behavioral response is initiated. What role then does the evolving response in the PN ensemble play in olfactory coding? There are several possibilities. It may be that the fixed point reached in the PN ensemble (Figure 3C and Figure 6B), as described for locusts [25], represents a state during which a change in the stimulus can easily alter the state of the PN network [42]. This sensitivity to a change in the stimulus would be transmitted quickly to the KCs of the MB. In addition, honey bees continue to ‘evaluate’ an odor long after a behavioral response occurs. When honey bees are tested with odors that are perceptually similar to but different from the conditioned odor, they show intermittent responses throughout the length of the stimulus [52]. This ‘indecisiveness’ implies continuous evaluation of the odor on the timescale of the AL ensemble. The later behavioral responses may be driven by reactivation of the MB due to a fluctuation in the AL output, or it could be driven by parallel processing of the AL output in brain centers outside of the MBs.

It may also be that the evolving PN response is related to plasticity, since the same octopamine-driven reinforcement signal is present in the AL and MB [53], [54]. Neural networks in both, AL [7], [55] and MB [48] change as a result of conditioning. In fact, associative conditioning shifts the population response maxima in the EN ensemble from initially ∼90 ms before conditioning, which is consistent with our results, to ∼200 ms after conditioning [22], which is the response maximum of the PN ensemble we report here. This might indicate that the ENs integrate information over a broader timeframe after conditioning. Moreover, after associative conditioning the ENs may indeed classify better than the AL, which would be consistent with theory [34], [35].

Finally, the MB output is also fast enough to potentially be combined with odor information streams from the AL that are sent in parallel to other areas of the brain. Some axon tracts emanating from the AL pass through or specifically target areas outside of the MB, such as the protocerebral lobes [56]. In addition fast output from the MBs could provide feedback to the ongoing computation in the AL itself. Such higher order feedback neurons were related to centrifugal system following the terminology of their analogs in vertebrates [19]–[21]. Centrifugal neurons were described in different insects e.g. in moth *Manduca sexta* and in cockroach *P. Americana*, where cell bodies of serotonin-immunoreactive centrifugal neurons were located in the AL cell clusters [57], [58]. In the honey bee the antennal lobe feedback neuron AL-1 [17], [18] or the ALF-1 neuron [16], were located at the ventral alpha-lobe of the MB, the region were we recorded EN activity. It connects the MB output retrograde with the AL. Indeed a functional feedback from KCs to PNs and local interneurons mediated by the beta-gamma-lobes is present in *Drosophila* [59]. Rapid odor classification at the MB output as we found it here would allow integrating higher order feedback into the ongoing odor computation of the AL.

## Supporting Information

Figure S1 **Electrode position and spike sorting.** To allow visualization of the electrode positions relative to the neuropiles, electrodes were dipped in a fluorescent dye (DiI, invitrogen.com) before positioning. After recording electrodes were removed, brains dissected and further on dehydrated as described in the method section. (**A**) To record activity of alpha-lobe extrinsic neurons the electrodes were inserted into the ventral region of the alpha-lobe (αL). (**E**) To record activity of projection neurons electrodes were inserted into the dorsal region (neck) of the antennal-lobe (AL). (**B** and **F**) High-pass filtered (800 Hz) differential extracellular recording channels. In our recordings from the ventral alpha-lobe and the dorsal antennal-lobe we obtained comparatively high spike signal amplitudes. Mean activity and standard deviation (SD) of the high-pass filtered channels were calculated. Thresholds for detecting events were always set above 3xSD. (**C** and **G**) Threshold crossing events were used to compute templates of spike waveforms which were subsequently used to assign individual spikes (semi-automatic spike sorting technique, in Spike2). (**D** and **H**) To ensure optimal and robust results of our spike sorting procedure we evaluated the first three Principal components of the related spike wave forms as a criterion of the sorting quality. Only spikes of separated clusters were used further on and interpreted as units. In the supplemental material of an earlier publication [22] we illustrated how EN activity can be differentiated from Kenyon Cell activity at the ventral alpha-lobe.
(TIF)

Figure S2 **Response detection example of a PN-unit to the presentation of 1-hexanol.** To detect significantly high firing rates for each neuron, we set a significance level q, and found the qth-percentile of the IFR distribution (cp. methods). The firing rate associated to q was used as a threshold beyond which the firing rates were deemed as significantly high. The blue line indicates the significance level q, which was set to 0.95%. (**left**) Mean instantaneous firing rate (IFR) averaged across 10 trials (grey line). The three seconds of odor stimulation were marked in light grey. IFRs crossing the threshold were marked using red dots. (**right**) Distribution function of the mean IFRs. Note, our test is rather conservative, since IFRs between 15 and 25 Hz might be related to the tonic activity, but were not extracted by the used threshold.
(TIF)

Figure S3 **Eight simultaneous recorded PN-EN couples (same color code) recorded out of eight different bees.** Out of the eight bees in which we recorded simultaneously PN and EN activity we chose one PN and one EN per bee (same color code). Out of the 10 repetitions per odor we calculated the mean latencies (individually color coded tick marks) for single PNs (**left**) and single ENs (**right**). The three rows correspond to the different odor stimuli. The grey line in each plot marks the related population response (normalized Euclidian Distance to 0). The dotted black line marked the stimulus onset. Red crosses indicate the significant threshold crossing of the related population response (significance level q>0.95). Red numbers indicate the population response latency (PL). The odor dependent latencies between the simultaneously recorded PN-EN couples out of each animal (bee 140–147) are drawn in the middle. If the latency is ‘empty’, either the PN or the EN showed no response. The same couple can show a positive latency (first PN than EN) for one odor whereas for another odor it can show a negative latency (first EN than PN). For example; in bee145 (magenta) 1-hexanol evoked a very early response in the PN (∼50 ms), 36 ms later in the same animal an EN follows to respond. Both neurons responded before their populations crossed the threshold. During the presentation of 2-octanone the PN did not respond, whereas the EN responded but after the population crossed threshold.
(TIF)
