# Supplementary figures and images for: Ensemble Response in Mushroom Body Output Neurons of the Honey Bee Outpaces Spatiotemporal Odor Processing Two Synapses Earlier in the Antennal Lobe
Source: PLoS One. 2012 Nov 29;7(11):e50322. doi: 10.1371/journal.pone.0050322 (PMC3510213; doi:10.1371/journal.pone.0050322)

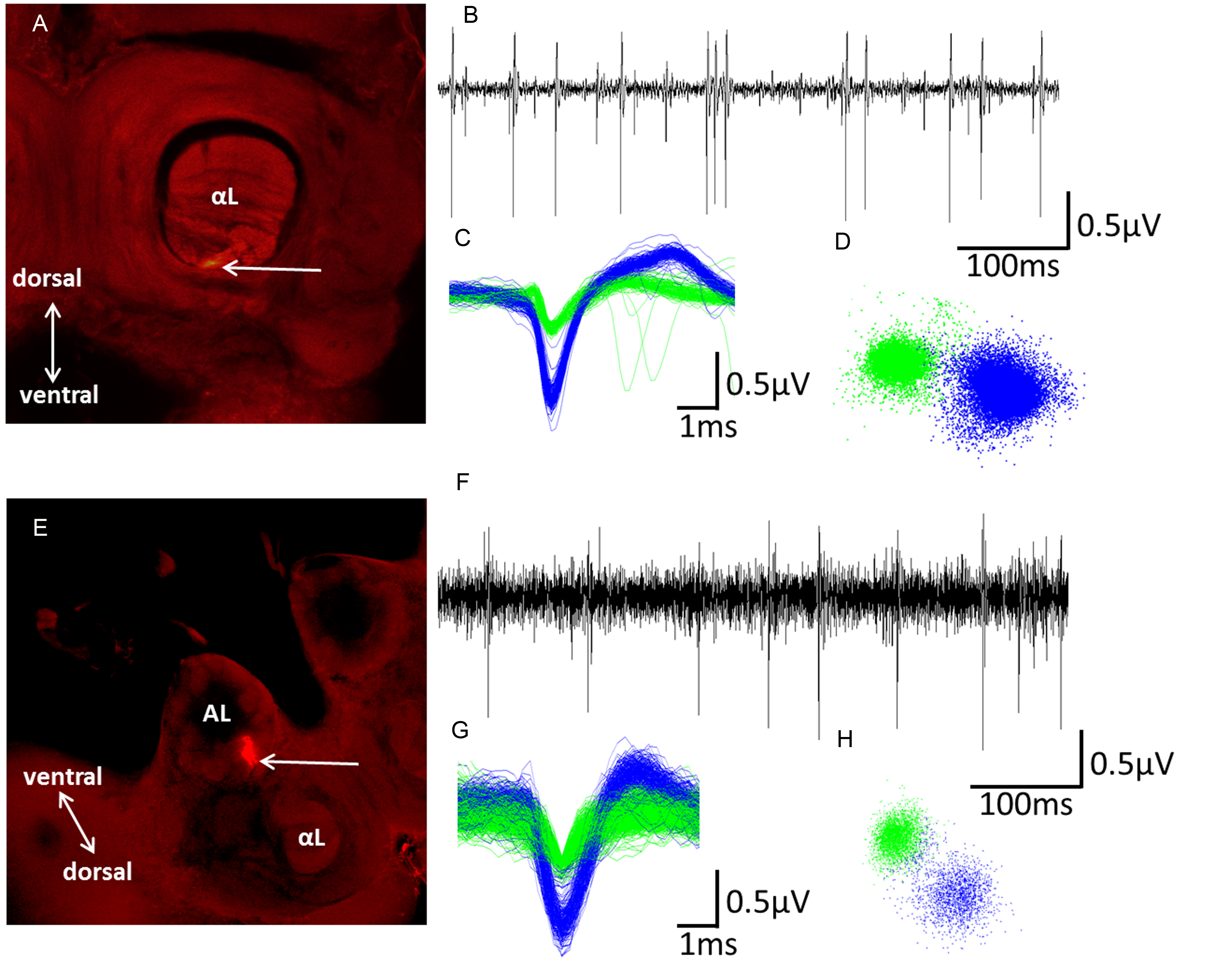

Supplement: Figure S1 — Electrode position and spike sorting. To allow visualization of the electrode positions relative to the neuropiles, electrodes were dipped in a fluorescent dye (DiI, invitrogen.com) before positioning. After recording electrodes were removed, brains dissected and further on dehydrated as described in the method section. (A) To record activity of alpha-lobe extrinsic neurons the electrodes were inserted into the ventral region of the alpha-lobe (αL). (E) To record activity of projection neurons electrodes were inserted into the dorsal region (neck) of the antennal-lobe (AL). (B and F) High-pass filtered (800 Hz) differential extracellular recording channels. In our recordings from the ventral alpha-lobe and the dorsal antennal-lobe we obtained comparatively high spike signal amplitudes. Mean activity and standard deviation (SD) of the high-pass filtered channels were calculated. Thresholds for detecting events were always set above 3xSD. (C and G) Threshold crossing events were used to compute templates of spike waveforms which were subsequently used to assign individual spikes (semi-automatic spike sorting technique, in Spike2). (D and H) To ensure optimal and robust results of our spike sorting procedure we evaluated the first three Principal components of the related spike wave forms as a criterion of the sorting quality. Only spikes of separated clusters were used further on and interpreted as units. In the supplemental material of an earlier publication [22] we illustrated how EN activity can be differentiated from Kenyon Cell activity at the ventral alpha-lobe. (TIF) [file pone.0050322.s001.tif]

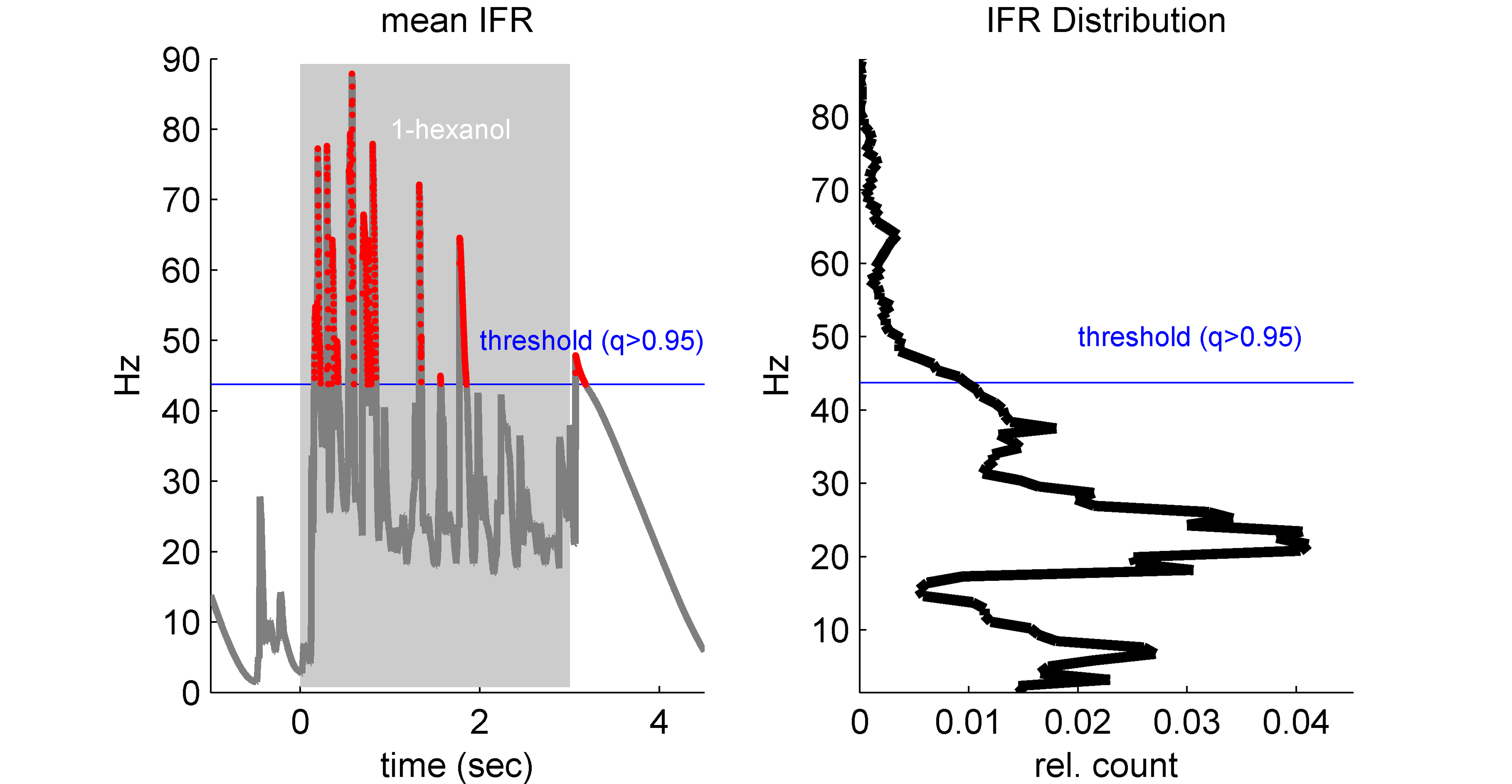

Supplement: Figure S2 — Response detection example of a PN-unit to the presentation of 1-hexanol. To detect significantly high firing rates for each neuron, we set a significance level q, and found the qth-percentile of the IFR distribution (cp. methods). The firing rate associated to q was used as a threshold beyond which the firing rates were deemed as significantly high. The blue line indicates the significance level q, which was set to 0.95%. (left) Mean instantaneous firing rate (IFR) averaged across 10 trials (grey line). The three seconds of odor stimulation were marked in light grey. IFRs crossing the threshold were marked using red dots. (right) Distribution function of the mean IFRs. Note, our test is rather conservative, since IFRs between 15 and 25 Hz might be related to the tonic activity, but were not extracted by the used threshold. (TIF) [file pone.0050322.s002.tif]

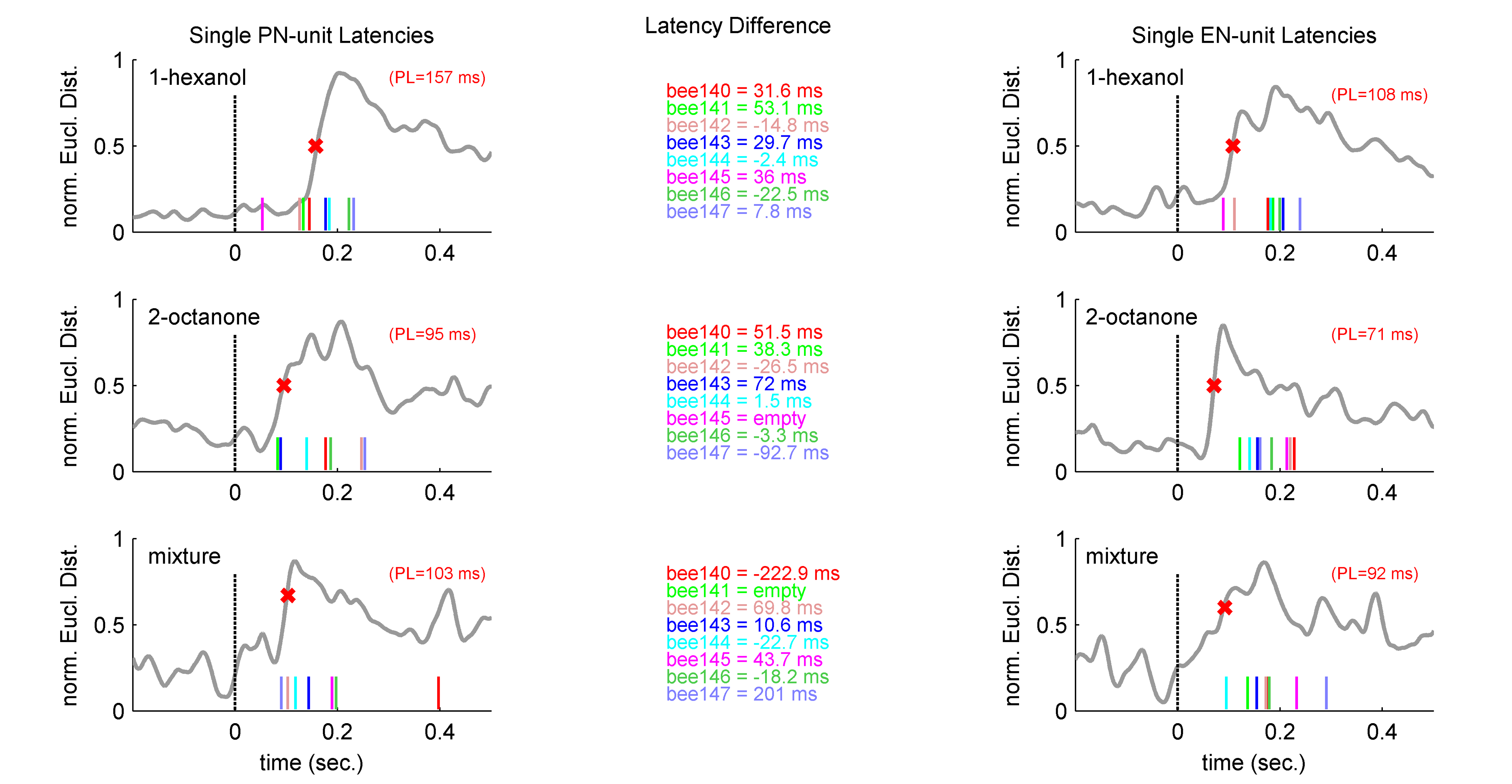

Supplement: Figure S3 — Eight simultaneous recorded PN-EN couples (same color code) recorded out of eight different bees. Out of the eight bees in which we recorded simultaneously PN and EN activity we chose one PN and one EN per bee (same color code). Out of the 10 repetitions per odor we calculated the mean latencies (individually color coded tick marks) for single PNs (left) and single ENs (right). The three rows correspond to the different odor stimuli. The grey line in each plot marks the related population response (normalized Euclidian Distance to 0). The dotted black line marked the stimulus onset. Red crosses indicate the significant threshold crossing of the related population response (significance level q>0.95). Red numbers indicate the population response latency (PL). The odor dependent latencies between the simultaneously recorded PN-EN couples out of each animal (bee 140–147) are drawn in the middle. If the latency is ‘empty’, either the PN or the EN showed no response. The same couple can show a positive latency (first PN than EN) for one odor whereas for another odor it can show a negative latency (first EN than PN). For example; in bee145 (magenta) 1-hexanol evoked a very early response in the PN (∼50 ms), 36 ms later in the same animal an EN follows to respond. Both neurons responded before their populations crossed the threshold. During the presentation of 2-octanone the PN did not respond, whereas the EN responded but after the population crossed threshold. (TIF) [file pone.0050322.s003.tif]
